# treedata.table: a wrapper for data.table that enables fast manipulation of large phylogenetic trees matched to data

**DOI:** 10.7717/peerj.12450

**Published:** 2021-11-26

**Authors:** Cristian Román Palacios, April Wright, Josef Uyeda

**Affiliations:** 1 Department of Atmospheric and Oceanic Sciences; Department of Earth, Planetary, and Space Sciences, Institute of the Environment and Sustainability; Center for Diverse Leadership in Science, University of California, Los Angeles, Los Angeles, CA, United States of America; 2 School of Information, University of Arizona, Tucson, AZ, United States of America; 3 Biology Department, Southeastern Louisiana University, Hammond, LA, United States of America; 4 Department of Biological Sciences, Virginia Polytechnic Institute and State University (Virginia Tech), Blacksburg, VA, United States of America

**Keywords:** Data.table, Evolution, Phylogenetics, Phylogenetic comparative analyses, R Package

## Abstract

The number of terminals in phylogenetic trees has significantly increased over the last decade. This trend reflects recent advances in next-generation sequencing, accessibility of public data repositories, and the increased use of phylogenies in many fields. Despite R being central to the analysis of phylogenetic data, manipulation of phylogenetic comparative datasets remains slow, complex, and poorly reproducible. Here, we describe the first R package extending the functionality and syntax of data.table to explicitly deal with phylogenetic comparative datasets. treedata.table significantly increases speed and reproducibility during the data manipulation steps involved in the phylogenetic comparative workflow in R. The latest release of treedata.table is currently available through CRAN (https://cran.r-project.org/web/packages/treedata.table/). Additional documentation can be accessed through rOpenSci (https://ropensci.github.io/treedata.table/).

## Introduction

**Figure 1:**
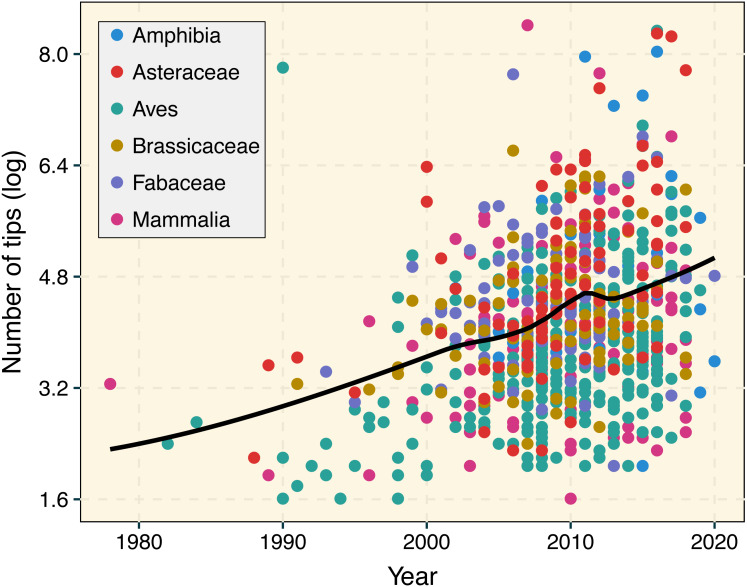
Temporal change in phylogenetic tree sizes between 1978 and 2020 based on 927 publications for different animal and plant groups. We used a LOESS smoothing to depict the temporal trend in tree size over time. Data was retrieved from the Open Tree of Life (Redelings et al., 2019) using the rotl R package (Michonneau, Brown & Winter, 2016). A linear regression that accounted for lineage identity indicated the significant increase in tree size over time (*R*^2^ = 0.2077, *p* < 0.001).

The number and size of published phylogenetic trees have exponentially increased over the years (Fig. 1; Smith et al., 2011; Fitzjohn et al., 2014; Smith & Brown, 2018). Ongoing biodiversity sequencing efforts have triggered the development of phylogenetic computational methods able to deal with datasets involving hundreds of thousands of taxa (McMahon et al., 2015). For instance, the early development of MAFFT (Katoh, 2002) significantly decreased computational times required to perform sequence alignment on molecular datasets with thousands of species. Similarly, RAxML (Stamatakis, 2006), PATHd8 (Tamura et al., 2012), and TreePL (Smith & O’Meara, 2012) greatly reduced computational times during the inference and absolute dating of phylogenetic trees including thousands of species. Given the unprecedented pace at which phylogenetic data is accumulating (Piel et al., 2000; Redelings & Holder, 2017), updating the current comparative phylogenetic workflow to cope with the increasing size of phylogenetic trees is now more critical than ever. Attention should be paid to the development of faster, computationally efficient, and more user-friendly implementations in R that further increase reproducibility. The R language (R Core Team, 2013) is now central to research utilizing phylogenetic comparative methods, and many essential packages and educational materials are made available using this language (Harmon, 2019). The latest release of treedata.table is available through CRAN (https://cran.r-project.org/web/packages/treedata.table/). More information about the treedata.table R package can be found in rOpenSci (https://ropensci.github.io/treedata.table/).

### A short description of data.table

treedata.table heavily relies on data.table, an R package that enables high-performance extended functionality for data tables (Dowle & Srinivasan, 2019). data.table is not only faster than other packages implemented in R, but also is significantly more efficient than tools in other languages such as Python and Julia (db-benchmark project, 2021). In addition to speed, data.table has a syntactic structure that is clear and simple to follow. Only three elements are basic to data.table’s primary function: DT[i, j, by]. First, the *i* section is used to specify the rows to be considered in filtering or subsetting operations. Second, the *j* section indicates the changes happening in the columns (*e.g.*, adding new ones, changing existing ones). Third, the by section is used to perform operations based on grouping variables. A brief but more exhaustive introduction to data.table can be found in the data.table’s vignette and wiki.

Data manipulation can be performed through numerous approaches in R. Each of these alternatives have their own particular advantages. For instance, packages in the tidyverse (*e.g.*, dplyr) are in general designed to increase readability and flexibility during data munging steps (Wickham et al., 2019a; Wickham et al., 2019b). Data wrangling in base R is largely standard and in general more stable over time than other approaches. Here, we focus on extending the functionality of data.table, a package that is generally faster and more concise than other approaches, for dealing with phylogenetic comparative datasets.

### The treedata.table workflow

treedata.table is a wrapper for data.table designed for phylogenetic analyses that matches a phylogeny to a data.table (Table 1). After an initial tree/data matching step, treedata.table continuously preserves the tree/data matching across data.table operations. treedata.table also allows users to run functions from other phylogenetic packages on the processed treedata.table objects. Below, we briefly explain the general workflow under treedata.table.

**Table 1:** Brief descriptions of the functions implemented in treedata.table. We list functions under eight different categories and provide a brief outline of their main uses.

Category	Function	Description
treedata.table object creation	as.treedata.table	Initial step of the workflow in treedata.table. Matches a character matrix (of class data.frame) to a single (of class phylo) or multiple trees (class multiPhylo)
Drop taxa from treedata.table objects	droptreedata.table	Drops taxa from a treedata.table object
Data manipulation	[	Performs data.table operations on an object of class treedata.table
Data extraction	[[	Extracts a named vector from an object of class treedata.table
	extractVector	Returns a named vector from a treedata.table object
	pulltreedata.table	Returns a character matrix or tree(s) from a treedata.table object
Run functions from other packages	tdt	Runs a function on a treedata.table object
Detect character type	detectCharacterType	Detects whether a character is continuous or discrete
	detectAllCharacters	Applies detectCharacterType over an entire character matrix
	filterMatrix	Filters a matrix, returning either all continuous or all discrete characters
Examine treedata.table objects	summary	Summarizes treedata.table objects by presenting the number of discrete and continious characters, missing values, and general changes to the original treedata.table object
	print	Print method treedata.table objects
	head, tail	Returns the first or last part of an treedata.table object
Inspect column/row names	hasNames	Row and column name check
	forceNames	Force names for rows, columns or both

(1) **Tree and character matrix matching:** Using the treedata.table package begins with creating a treedata.table object. as.treedata.table function matches the tip.labels of the phylogeny to a column of names in the data.frame.

(2) **treedata.table** **operations:** two main functions allow users to make changes to treedata.table objects. Changes are reciprocal between trees and data.

(A) **Explicitly dropping taxa:** Taxa in treedata.table objects can be dropped using the droptreedata.table function. Dropped taxa results are removed from the character matrix and trees.
(B) **Data operations:** The most powerful functionality of treedata.table is related to functions calling data.table. The [ function, taking the same arguments as the analog function in data.table, can be used to subset rows, select, and/or compute statistics on columns in the character matrix of the treedata.table object (DT[i, j, by]). Operations changing the number of rows in the character matrix will also affect the corresponding taxa in the tree.

(3) **Data extraction from** **treedata.table** **objects:** Users can independently extract trees and character matrices from treedata.table objects using the pulltreedata.table function. The $ operator is also a valid alternative to pulltreedata.table. Two additional functions ([[ and extractVectors) can be used to extract named vectors from treedata.table objects. These operations streamline formatting of data into the various different input requirements of R functions from other phylogenetics packages.

(4) **Using external functions in** **treedata.table** **objects:** the tdt function enables users to easily run external functions on treedata.table objects directly. Specifically, tdt passes data and tree attributes from a given treedata.table object as arguments to functions implemented in other packages.

(5) **Additional functions**: treedata.tree includes additional functions to detect and filter character matrices by character types (continuous or discrete; detectCharacterType, detectAllCharacters, and filterMatrix). Other functions can be used to examine (head, tail, print) and describe (summary) objects of class treedata.table. Finally, two additional functions can be used to inspect and force column and row names in character matrices (hasNames, forceNames).

### Using treedata.table

This brief step-by-step tutorial is based on treeplyr’s *Anolis* example data, including 100 tips and 11 characters (see also Appendices S1–S2):

library(treedata.table)

data(anolis)

To use all the functionalities in treedata.table, we first construct a treedata.table object using the as.treedata.table function, which performs an exact name match between the tip labels of the tree and the column in the dataset with the most matches.

td <- as.treedata.table (tree = anolis$phy, data = anolis$dat)

The resulting object can be inspected using the summary(), head(), tail(), and print() functions. For instance, we can see a description of the treedata.tree object using the summary() function:

summary(td)

Next, we can perform data manipulation steps on the resulting treedata.table object. For instance, we can extract the SVL column (snout-vent length) using the $ function and [ operator, as follows:

td$dat[,’SVL’]

A named vector of the same trait (SVL) can also be extracted using td[[”SVL”]] or extractVector(td, ’SVL’). However, extractVector further supports extraction of multiple traits. For instance, the following code will extract two named vectors: one for SVL and another for ecomorph.

extractVector(td, ’SVL’,’ecomorph’)

The real power in treedata.table is in co-indexing the tree and table based on functions from data.table. We can use data.table syntax to subset the treedata.table object and include only the first representative from each ecomorph in the *Anolis* dataset.

td[, head(.SD, 1), by = ”ecomorph”]

We can also subset the *Anolis* dataset to include a single species per ecomorph and island:

td[, head(.SD, 1), by = .(ecomorph, island)]

Furthermore, we can create a new variable summarizing SVL+hostility for only Cuban anoles:

td[island == ”Cuba”, .(Index = SVL + hostility)]

While the options for data manipulations are infinite, the matching between the tree and data attributes is always constant. Finally, users can pass data and trees in treedata.table objects as arguments to functions in other packages. For instance, below we use the tdt function in treedata.table to fit a continuous model of trait evolution for SVL in geiger (Harmon et al., 2008; Pennell et al., 2014):

tdt(td, fitContinuous(phy, extractVector(td, ’SVL’), model=”BM”))

All the functions explained above can handle multiple trees. For instance, below we fit the same model of continuous trait evolution on SVL based on a multiPhylo tree for the *Anolis* dataset:

trees <- list(anolis$phy,anolis$phy)

class(trees) <- “multiPhylo”

td <- as.treedata.table (tree=trees, data=anolis$dat)

tdt(td, fitContinuous(phy, extractVector(td, ‘SVL’), model=”BM”))

The introductory vignette to treedata.table (https://ropensci.github.io/treedata.table/articles/AA_treedata.table_intro_english.html, https://ropensci.github.io/treedata.table/articles/AB_treedata.table_intro_spanish.html) contains further information on the functions outlined above and in Table 1.

### Computational performance

#### Alternatives to treedata.table

Keeping trees and data objects separated in the R environment is a standard practice. Changes to trees and data are typically performed independently using a combination of functions implemented in ape (Paradis & Schliep, 2018), base (R Core Team, 2013), data.table (Dowle & Srinivasan, 2019), or in the tidyverse (Wickham et al., 2019a; Wickham et al., 2019b). However, to our knowledge, treeplyr (Uyeda & Harmon, 2020) and tidytree (Yu, 2021), both based on dplyr (Wickham et al., 2019a; Wickham et al., 2019b), are to our knowledge, the only packages that are able to perform simultaneous operations on combined tree/data objects in R (Table 2). We note that while data.table, treeplyr, and dplyr share similar functionalities, their philosophy and syntax are strikingly different. Differences between these packages ultimately relate to “source” package they rely on (*i.e.*, data.table or dplyr). For instance, although data.table uses shorter syntax relative to dplyr, the pipe operator and the use of verbs in dplyr makes this later package more intuitive and easier to debug. Therefore, although our package treedata.table only extends the functionality of data.table s into the phylogenetic comparative workflow, this largely unexplored framework in the field will enable users to take advantage the speed and syntax of that is inherent to data.table.

**Table 2:** Functions in different R packages (including treedata.table) with similar functions on matched tree/data objects.

Package	Function	Tree/data-matched object manipulation	Reference
treedata.table	as.treedata.table	data.table syntax	This study
geiger	treedata	Not supported	Harmon et al. (2008), Pennell et al. (2014)
tidytree	treedata	dplyr verbs after using tibble::as_tibble()	Yu (2021)
treeplyr	make.treedata	dplyr verbs	Uyeda & Harmon (2020)

#### Methods

We used the microbenchmark (Mersmann, 2019) function under default parameters to compare the performance of functions in treedata.table to other packages (Appendix S3). First, we compared the performance in the initial tree/data matching step between treedata.table and treeplyr (treedata.table::as.treedata.table () and treeplyr::make.treedata()). We simulated trees with 10, 40, and 100 tips using rtree function in ape (Paradis & Schliep, 2018). Additionally, we generated random character matrices (50 discrete and 50 continuous traits) matching 90% of tips in the tree. Second, we compared the performance of data operations in treedata.table relative to data.table (Dowle & Srinivasan, 2019), base (R Core Team, 2013), treeplyr (Uyeda & Harmon, 2020), and dplyr (Wickham et al., 2019a; Wickham et al., 2019b). This time, we simulated trees with 1000, 10000, and 500000 tips using the rtree function in ape. Again, we generated random character matrices (50 discrete and 50 continuous traits) matching 90% of tips in simulated trees. We compared the performance of treedata.table::[, data.table::[ treeplyr::%>%, dplyr::%>%, and the equivalent functions in base when (1) subsampling the full dataset for rows matching a single level in one discrete character, and (2) estimating the sum and mean of two continuous traits based on the groups of a second discrete character. In data.table syntax for this process would be:

td$dat[Disc1 == ”A”, .(sum(Cont2), mean(Cont3)), by = Disc10]

#### Results

**Figure 2:**
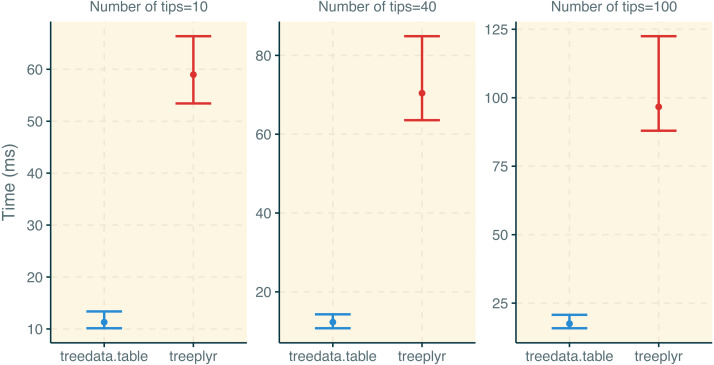
Results for the treedata.table microbenchmark during tree/data matching steps. Estimates of the timing during the tree/data matching steps under treedata.table are shown in relation to treeplyr. We show median and lower/upper quartiles times for the performance of each package.

**Figure 3:**
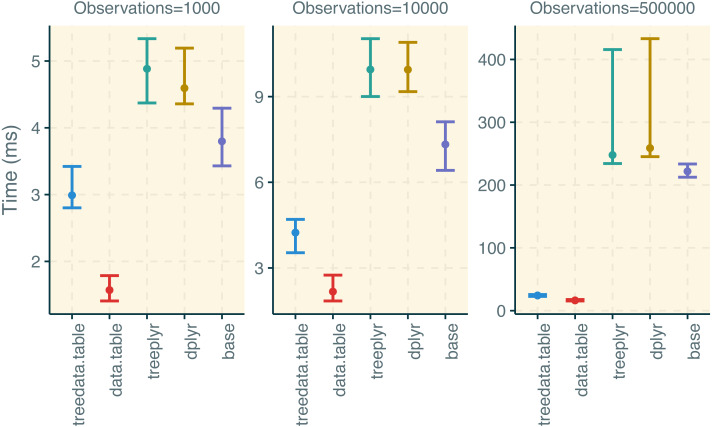
Results for the treedata.table microbenchmark during data manipulation. We compare the performance of treedata.table against data.table, base, treeplyr, and dplyr. We show median and lower/upper quartiles times for the performance of each package.

treedata.table was >400% faster than treeplyr during the initial data/tree matching step (Fig. 2). For instance, combining a dataset with 10 tips to a character matrix of 40 traits (10% of unmatched tips), as.treedata.table takes an average of 12.314 ms (range = 8.100–27.479 ms) relative to the 64.198 ms that were needed in treeplyr (range = 48.407–166.328 ms). Differences in the performance between these two functions also scale with the number of taxa. Next, we examined the performance of data operations in treedata.table relative to data.table, base, treeplyr, and dplyr (Fig. 3). We found that the simultaneous processing of phylogenetic trees in treedata.table’s compromised the speed of our package by 90% relative to data.table. However, data manipulation in treedata.table (which simultaneously processes phylogenies) is still significantly faster than in other commonly used packages for data manipulation only, such as base (>35%), treeplyr (>60%), and dplyr (>90%). The higher speed performance of treedata.table relative to other functions also increases with the size of the dataset.

### Current limitations of treedata.table

The current release of treedata.table can handle phylo and multiPhylo objects. A single character matrix is shared across all the trees in the treedata.table object. Additionally, all the trees and the only character matrix in the same treedata.table object are forced to have the same tip-level sampling. We acknowledge that partial tree/data matching is desirable in some situations. For instance, users may be interested in performing analyses on trees that, despite having different tip-level sampling, partially overlap with a common character matrix. Similarly, users may be interested in using multiple character matrices instead of only one. Future releases of the treedata.table package will focus on relaxing some restrictions on the tree/data matching.

## Conclusions

Here we describe the first R package that extends the functionality and syntax of data.table for performing operations in phylogenetic comparative datasets. We also note that treedata.table significantly improves the speed of the analytical workflow when compared to alternative methods for manipulating phylogenetic comparative data. treedata.table is expected to increase code reproducibility while simplifying the complexity of scripts. Finally, data manipulation in treedata.table, which is significantly faster than in other commonly used packages, will allow researchers to quickly perform data manipulation on large datasets without requiring outstanding computational resources.

## Supplemental Information

10.7717/peerj.12450/supp-1 Supplemental Information 1 Getting started with the treedata.table package

10.7717/peerj.12450/supp-2 Supplemental Information 2 Breve introducción al paquete treedata.table Getting started with the treedata.table package in Spanish.

10.7717/peerj.12450/supp-3 Supplemental Information 3 R script used to perform performance comparison between treedata.table and other methods

10.7717/peerj.12450/supp-4 Supplemental Information 4 R script used to generate Fig. 1

## Additional Information and Declarations

### Data Availability

The following information was supplied regarding data availability: The treedata.table is available at https://ropensci.github.io/treedata.table/index.html.
